# Analysis of an Online English Teaching Model Application Based on Improved Multiorganizational Particle Population Optimization Algorithm

**DOI:** 10.1155/2021/6232987

**Published:** 2021-12-16

**Authors:** Fang Wang

**Affiliations:** Jiangsu Lianyungang Higher Vocational and Technical School of Traditional Chinese Medicine, Lianyungang, Jiangsu 222007, China

## Abstract

This paper uses an improved multiorganizational particle population optimization algorithm to conduct an in-depth analysis and study of an online English teaching model and uses the altered model for practical applications. The model building elements are extracted from it for the initial construction of a blended learning model of English-speaking teaching in junior high school. The main purpose of the first round of action research is to test the rationality of each element of the model, the main purpose of the second round of action research is to refine the model links and improve the operability of the model, and the main purpose in the third round of action research is to test the perfected model and explore the model. The main purpose of the third round of action research is to test the refined model and explore the application suggestions of the model. After the three rounds of action research, we finally obtained a more mature blended learning model for teaching English as a foreign language in junior high school. Mainly through the comparison of the pre- and posttest scores of English speaking of the experimental subjects and the comparison of the pre- and posttest data of the relevant questionnaires, the following experimental conclusions were drawn: adopting the blended learning-based speaking teaching model can effectively improve students' interest in learning English, their attitudes and their English speaking skills including pronunciation, phonetic intonation, conversational communication, and oral expression and can enhance students' group cooperation and communication ability, independent learning ability, evaluation awareness, and ability. This single-guided learning mechanism can effectively avoid the shocks that are easily caused by the dual-guided role of traditional PSO. The dimensional learning strategy constructs a learning paradigm for each particle by learning from each dimension of the individual optimal position of the particle to the corresponding dimension of the group optimal position, respectively. Dimensional learning is formally integrated into the learning paradigm only if it can improve the fitness of the paradigm so that the dimensional learning strategy can avoid the phenomenon of degradation of the learning paradigm and the phenomenon of “two steps forward, one step back.” In the dimensional learning strategy, since each particle learns from best, although it has a strong exploitation capability, it may cause all particles to converge to best quickly, making the algorithm converge prematurely.

## 1. Introduction

With the development of information technology, traditional classroom teaching can no longer meet the diverse needs of teachers and students in terms of time and location, while online teaching breaks the time and space constraints of traditional classroom teaching and creates an environment where students can learn even outside the classroom. For this reason, the addition of information technology has led to dramatic changes in the format of teaching, the types of teaching resources, and student learning behaviours. A new teaching model that combines the advantages of online teaching and traditional offline teaching, the hybrid teaching model, has emerged [1]. Offline teaching and online teaching have their advantages and limitations and cannot replace each other. Hybrid teaching mode is a teaching model that combines the advantages of traditional offline teaching and emerging online teaching [2]. This model makes educational resources richer and learning activities more diverse, especially with the popularity of mobile terminals such as smartphones and iPods and the rapid development of some excellent APPs, which allows students to learn conveniently and quickly anytime and anywhere with a single click on their mobile phones, breaking the limitations of time and space for students in traditional teaching. In recent years, the smart campus is being rapidly built and promoted, education data is growing exponentially, and education is rapidly moving into the era of big data [3]. Teaching is a dynamic, interactive, and continuous process, which is bound to generate a large amount of timely and diverse data. This data serves as a basis for judging student learning, which in turn responds to teachers' teaching standards. Therefore, mining and analysing the data is necessary. Learning analytics is precisely the measurement, collection, analysis, and reporting of educational data, using different techniques to analyse learning data to discover student characteristics and understand teaching and learning. Common techniques include statistical analysis, cluster analysis, and correlation analysis. With the popularity of smart campuses, increased educators and researchers are coming to focus on students' learning behaviours. With the development of information technology in education, learners have increased ways to acquire knowledge and have higher requirements for education, with more focus on whether teachers can tailor and personalize their teaching. Personalized teaching is a new form of teaching that has emerged in recent years, and it is a way to achieve good teaching results by stimulating learners to think and develop [4]. The teaching process will be optimized for the needs of each learner and is closely related to the learner's interests. In the context of education informatization, education big data, and personalized teaching, the blended teaching model has emerged. It is of strong practical significance to analyse students' learning behaviour under the blended teaching model. Dimensional learning can only be formally integrated into the learning paradigm on the premise that it can improve the fitness of the paradigm. Therefore, the dimensional learning strategy can avoid the phenomenon of learning paradigm degradation. To gain a deeper understanding of the teaching of blended teaching mode and analyse students' learning behaviours under the blended teaching model, this study classifies students with the characteristics of learning behaviours, explores the correlation between learning behaviours and academic performance, and identifies the factors affecting learning behaviours. It is applied in practice to make up for the lack of research on learning behaviour in blended learning and teaching.

Particle swarm optimization algorithms are the primary choice for optimization problems due to less memory required, easy implementation, faster convergence, and the ability to provide better performance on different benchmark test functions and engineering problems. Particle swarm optimization algorithms are population-based iterative algorithms and the requirement of many evaluation functions for computation makes particle swarm optimization algorithms inappropriate for optimization areas where fitness computation is time-consuming. In solving complex problems with multiple extremes, the standard particle swarm algorithm tends to fall into local extremes, resulting in premature convergence without finding the optimal value of the entire search space; when the dimensionality of the search space is high, the algorithm has poor search capability [5]. These drawbacks have limited the wider application of particle swarm optimization algorithms. Therefore, speeding up convergence and avoiding getting into local extremes have become two of the most important and attractive goals in the research of particle swarm optimization algorithms. Exploration and exploitation are the two main search mechanisms in the search for optimal solutions in particle swarm optimization algorithms. To achieve fast convergence and avoid getting into local extremes, one must balance the exploration and exploitation capabilities of the population [6]. Exploration searches the region of the search space with potentially optimal solutions; more refined searches performed in the vicinity of the population optimal solution. Overemphasis on exploration will lead to a decrease in convergence by wasting search time in nonoptimal regions. On the other hand, overemphasis on exploitation will reduce the diversity of the population in the search process, which may be the population caught in the local optimal region. Therefore, how to balance the exploration and exploitation capabilities in the search process is a question worthy of deeper investigation.

In this paper, a two-population particle swarm optimization algorithm with a dimensional learning strategy is proposed from the perspective of effectively discovering and preserving good information in each dimension of the population, and the ability of the proposed algorithm to balance exploration and exploitation intensity is discussed. The particle swarm optimization algorithm is a heuristic stochastic search algorithm, and this stochastic search mechanism lacks certainty and does not guarantee that the algorithm can necessarily find the optimal solution. Online learning platforms provide students with rich resources, such as text, images, audio, video, and animation, but these resources mostly presented directly, the organization of resources is confusing, and the content and quality need to be further optimized. This leads to the phenomenon of students' knowledge being lost in the face of numerous learning resources. Again, there is a lack of relevance and adaptability. Although student autonomy has been increased, the content and path of learning remain neat and uniform for everyone, and the test questions are fixed.

### 1.1. Status of Research

How to break the shackles of traditional teaching methods and achieve the personalized development of students has become a major dilemma facing education in China at present [7]. With the development of big data, cloud computing, and artificial intelligence technology, the way of learning, teaching, and cognition is undergoing radical changes. Especially the development of big data in education makes the collection and deep analysis of educational data possible and makes the sharing of educational resources a reality [8]. How to be able to collect and integrate data and use the data to discover the hidden behind it becomes an important way to enhance the core competitiveness of education, and the whole society has entered the era of big data, helping particles escape from local extrema. TSLPSO reduces the pressure of DLPSO adaptability assessment by introducing dimensional learning subgroups. The education field has set off a boom of promoting education reform and development based on big data in education, and the research on big data has shown an unprecedented development momentum [9]. At the level of applied research, a personalized adaptive learning platform based on big data has become the focus of research. Adaptive learning platforms collect student interaction data based on big data technology and dig deeper and analyse them for learning, providing students with personalized learning services according to the individual differences of learners, which have taken root in many countries and developed into personalized adaptive learning platforms [10]. The US Adaptive Learning Platform is one of the most mature adaptive learning platforms at present. After decades of research, it integrates the research results of psychology, measurement, cognitive learning theory, and intelligent learning systems and pioneers the design and application of personalized services for big data in education. By collecting students' online learning data, it can accurately predict and analyse students' strengths, weaknesses, learning interests, cognitive levels, and participation levels and personalize learning contents. It not only meets the needs of students to a great extent but also brings great convenience to teachers, parents, and school administrators in assessment and management [11]. At this stage, China has also made many useful attempts in the theory and practice of adaptive learning, but most of the research remains at the theoretical level, and there are few applications and practices in this area, and there is no mature adaptive learning platform yet. Therefore, through the study of Newton's adaptive learning platform in the United States, it can inspire the development of adaptive learning platform and personalized teaching in China.

The neural network uses a multilayer structure where the basic processing unit is a neuron in each layer of the network and contains both linear superposition and nonlinear activation as two types of computation, so mathematically speaking, a neural network is a structure with nesting that can be used to describe highly and nonlinear mapping relationships [12]. Neural networks are not all smooth sailing, it has experienced many troughs since it was proposed, but each time it came out of the trough was when it made a breakthrough and gradually became the core algorithm of current artificial intelligence [13]. Its main feature is the backpropagation algorithm, thus significantly reducing the time required for model training. Subsequently, scholars have never stopped their research on BP neural networks. Because it did not have the problem of gradient disappearance like the neural network that used more layers of the network in the environment with a relatively small amount of data at that time, this algorithm with the characteristics of simplicity and efficiency, fast training speed, and applicable to small sample size once overtook the deep neural network to become the mainstream of machine learning.

It has attracted many scholars and researchers to conduct in-depth research on it, thanks to its relatively simple concept, relatively easy implementation, and better global search capability when solving some more complex optimization problems, such as when the objective function is multipeaked, nonlinear, and nonderivative. Nowadays, the PSO algorithm has been applied to many engineering practices such as pattern recognition, intelligent robotics, and signal processing. The improved algorithm is used to solve the selection of parameters in the training process of machine learning, to improve the model performance and make the BP neural network in the initialization stage be in a relatively low loss for training, and to achieve the training requirements faster and better model and also can make the SVM model have better hyperparameters and the model after training have better results.

## 2. Improved Multiorganizational Particle Population Optimization Algorithm for Online English Teaching Model Applications

### 2.1. Improved Multiorganizational Particle Population Optimization Algorithm Design

The topology of a population defines the way information is shared and information has interacted between particles. Particle swarm algorithms based on neighbourhood topology control the exploration and exploitation capabilities of the algorithm according to the different information-sharing mechanisms between particles. A new information flow mechanism is proposed to update the position of each particle, namely, the full information particle swarm optimization algorithm (FIPS) [14]. The FIPS algorithm uses a weighted average of the individual optimal positions of all neighbouring particles to update the position of a given particle. The unified particle swarm optimization (UPSO) algorithm is proposed, which uses the optimal experience from both local and global neighbours to control the exploration and exploitation capabilities of the population. A dynamic neighbourhood learning particle swarm optimization (DNLPSO) algorithm is proposed, where exemplar particles are selected from the optimal positions of neighbouring particles including itself so that the velocity of a particle may be influenced by both the historical experience of its neighbours and its own historical experience; when the population perceives that the search has stalled, the population changes the flow of information. When the fitness of particle *i* does not improve within K generations, the particle is judged to be in stagnation, at which point the particle updates its neighbours by linking a new particle [15]. The particle uses a betting round selection strategy (based on particle fitness ranking) to select a new neighbour. In this way, underachievers who lack practice must complete as many as eight question sets or more, while students with stronger learning ability may complete adaptive follow-up after one question set.

Analysis of the PSO algorithm formulation shows that the particle flight trajectory is influenced by its own flight experience (individual optimum) as well as the global optimum information in the early stages and converges to the global optimum in the later stages [16]. This suggests that there is a better global optimum information point that will be able to guide the particle towards a better solution space. To obtain better global optimum information, the HPSO algorithm is proposed to achieve the position update by performing multiple Corsa mutations on the optimal particles. Most of the current mutation strategies for optimal particles pick all dimensions or randomly pick some dimensions for mutation; for high-dimensional complex functions, the computation of their fitness will be inefficient due to the interference between dimensions, which causes some dimensions to become better but be masked by other dimensions that become worse; compared to multiple mutations, the dimension-by-dimension mutation is more efficient. The obtained variational solutions are usually better [17]. Based on this, a variational strategy of dimension-by-dimension backward learning of the centre of gravity for the best particles is proposed, where dimension-by-dimension variation can reduce interdimensional interference and backward learning of the centre of gravity can expand the search space, increase the diversity of populations, and improve the convergence accuracy. However, the computational overhead of dimension-by-dimension backward learning is large, and it is not suitable for all particles to be mutated dimension by dimension:

(1) $$\begin{array}{c} Gbest_{i}=3\times k\times M_{i}+Gbest_{j}. \end{array}$$

**Figure 1:**
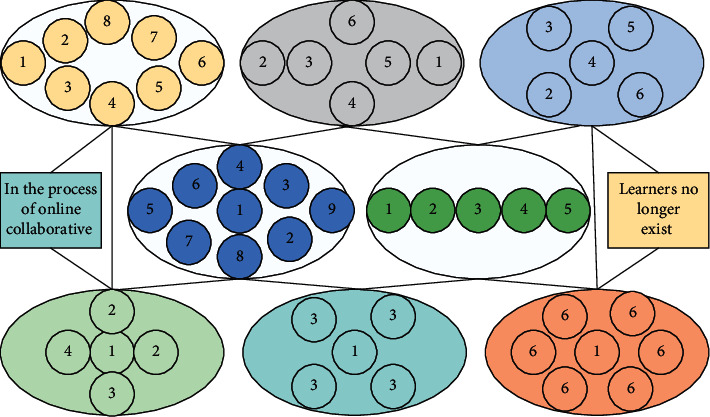
Framework of improved multiorganizational particle population optimization algorithm.

With the advent of the network era and the transformation of learning tools, methods, and resources, a more suitable connectivism for the digital information age is proposed based on behaviourism. Among them, connectionist learning theory believes that the starting point of all knowledge and learning is the individual, and the individual's knowledge can form a network, and in the process of learning and sharing knowledge, the nodes of the connection network and the nodes of other individuals, organizations, or institutions collide and interact with each other, and the connection of old and new nodes makes the continuous expansion of the individual's original network form a larger knowledge network. In the process of online collaborative learning, learners no longer exist as individual individuals but form learning groups through their will and rational choice and complete teaching tasks with learning groups as the basic unit. The learner's knowledge network collides and interacts with the knowledge networks of other members of the learning group, jointly expanding the original knowledge network, improving the learner's knowledge framework system, and enhancing the effect of online collaborative learning, as shown in Figure 1.

From the above example, we can see that when the dimensional learning strategy constructs a learning paradigm, each time the dimensional status of the temporary paradigm is updated, it is compared with the current learning paradigm, and if it is better than the current learning paradigm, the learning paradigm is updated to the current temporary paradigm; otherwise, the current learning paradigm is not changed and the learning process continues for the next dimension. The effect of face-to-face oral English teaching has been greatly improved. After class, dialogue exercises are arranged through the wing class network, and dialogue exercises are continued through the WeChat group cooperation group to achieve language review, consolidation, extension, and expansion and train students to speak and use English after class, which are good speaking habits:

(2) $$\begin{array}{c} Div=\frac{1}{n}\overset{m}{\underset{i=1}{\sum }}\sqrt{\sum_{j=1}^{m}{\left(x_{i,j}+x_{j}\right)}^{2}},\\ x_{j}=\frac{\sum_{j=1}^{m}{\left(x_{i,j}+x_{j}\right)}^{2}}{n}. \end{array}$$

We give plots of single-peaked and multipeaked functional diversity. The dimensionality of the search space in the diversity validation experiment is 30 dimensions, the function is evaluated 300,000 times, and the population size is 20. It is seen that the integrated learning subpopulation diversity is higher than the diversity of the dimensional learning subpopulation, and the diversity of the whole population lies between the two subpopulation diversities. The results from the diversity show that the dimensional learning subpopulation maintains a smaller population diversity and therefore converges quickly. As expected, the integrated learning subpopulation maintains a higher group diversity because there is no information as a central guiding direction, so the group does not converge to a smaller extent quickly. The balance of exploration and exploitation provided by the dimensional learning subgroup in collaboration with the integrative learning subgroup resulted in an intermediate diversity ranking for the whole group, indicating that the integrative learning strategy we introduced did increase the diversity of the group. Thus, the results of the diversity comparison validate the design expectation that the dimensional learning subgroup tends to local exploitation, while the integrated learning subgroup tends to global exploration. The interaction and cooperation of the two subpopulations ensure global search and fast convergence of the population:

(3) $$\begin{array}{c} f_{7}\left(x\right)=\overset{n}{\underset{i=1}{\sum }}\left[x_{i}^{2}+10\;\sin\left(2\tau x_{i}\right)-10\right],\\ f_{16}\left(x\right)=\sum_{i=1}^{n}\left[a_{i}x_{i}^{2}-10\;\sin\left(2a_{i}\tau x_{i}\right)+10\right]. \end{array}$$

**Figure 2:**
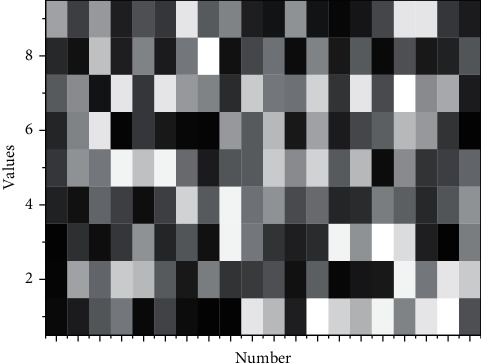
Contour plot of the function.

It is difficult to find its optimal solution for most of the algorithms, and when the dimension of the search space is higher than 3, it can be considered as a multipeaked function (Figure 2). Therefore, most of the algorithms have unsatisfactory results on functions. Although the optimal solution for the 3 functions out of 5 single-peaked functions is found, one more than the proposed one, it performs the worst on the function. Even though only the dimensional learning strategy was used, it still achieved the optimal result on the noise function. Overall, the algorithm shows better robustness on the single-peaked functions (i.e., the first 5 benchmark functions) and thus obtains a smaller sum than the champion algorithm. Multipeaked functions contain multiple locally optimal solutions, which may lead to premature convergence of the PSO algorithm. It is difficult to find the global optimal solution of the function with conventional PSO because the problem has many deep local optima far from the global optimal solution. The nodes of organizations or institutions collide and interact with each other, and the connection of new and old nodes makes the original network of individuals continue to expand and form a larger knowledge network. Once a particle of classical PSO is trapped in a deep local optimum, it is difficult to escape. The improved multiorganizational particle population optimization algorithm has better resistance to the local optimum trap and thus achieves a high accuracy of 3.82E-04. The other 5 bodies, however, cannot converge to the global optimum effectively, and the worst result is 2.38 E+03. Functions are very complex multipeaked functions with many local extrema. For this problem, algorithms that maintain better diversity tend to produce better results. The algorithm achieves the optimal solution on both functions, 0. This is due to the dimensional learning strategy and the integrated learning strategy used in the algorithm TSLPSO, which effectively enhances the diversity of the particle search while improving the convergence accuracy.

The users have different rating values for the target object, so a weighted average of the ratings is taken as the prediction result, and the weights can be obtained from the similarity between similar users and the target user:

(4) $$\begin{array}{c} R_{ik}=\mu_{ui}-\frac{\sum_{i,j}P\left(U_{i}U_{j}\right)}{\sum_{i,j}P{\left(U_{{}_{i}}^{2}U_{{}_{j}}^{2}\right)}^{2}}. \end{array}$$

Matrix decomposition is the decomposition of a matrix into the product of two or more matrices. Matrix decomposition-based recommendation algorithms are used to build user rating prediction models by modelling known user ratings of objects, which are mainly applied to two scenarios: rating prediction and TOP-N recommendation. Currently, the widely used matrix decomposition-based recommendation algorithm is the number of hidden factor models (LFM). The basic idea of LFM is to link user interests and items through implicit features, that is, the existence of similarity. The earliest appearing hidden factor model is the singular value matrix decomposition model, referred to as SVD, which is calculated as

(5) $$\begin{array}{c} R_{M\times N}=U_{M\times N}\sum M\left(M\times N\right). \end{array}$$

According to Wittrock, the king of generative learning theory, the essence of learning is a process of active construction and generation of meaning, in which learners construct meaning by actively establishing relationships between external stimuli and their original cognition. This construction of “special associations between external information and existing knowledge and experience” is called generation. The basic assumption of generative learning theory is that “the human brain is not a passive recipient of information, but an active constructor” [18]. In other words, the learner is not a passive recipient, but an active participant in the learning process, actively constructing a meaningful understanding of the information in the surrounding environment. However, without attention, motivation, and existing knowledge in memory, the construction of relationships between existing knowledge and new information will not occur. The comprehensive learning subgroup maintains a high group diversity, because there is no information from the central guide, so the group will not quickly gather into a smaller area. By assimilating Plus's information processing theory, Wittrock proposes the information processing process of the generative learning model: attention, motivation, prior knowledge experience, and generation. Attention is the directional factor that guides the generative process, which points the generative process to prior knowledge and experience. Motivation refers to the desire to actively generate these two connections and to attribute the effectiveness of generating them to the degree of one's effort; prior knowledge and experience include existing concepts, reflective cognition, abstract knowledge, and concrete experience, and the learner's prior cognitive structure and level are extremely important in generating the meaning of things; generation refers to the intrinsic connections that form new knowledge and the connections between new knowledge and existing experience.

### 2.2. Design of Online English Teaching Model Applications

**Figure 3:**
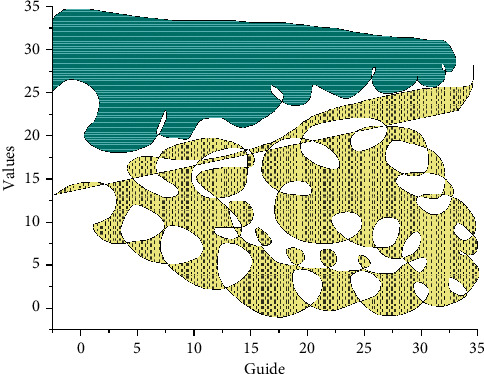
Diagrammatic representation of the constructive learning environment design model.

Constructivism assumes that students' knowledge is constructed based on their prior knowledge experiences and cognitive structures and is actively acquired through meaningful construction. The meaningful construction of knowledge requires interaction with the external environment and the use of other aids from certain situations, for example, with the help of other people including teachers, classmates, and peers, using the necessary learning materials and learning resources. Constructivist learning theory emphasizes the cognitive dynamics, the contextual nature of learning, the importance of resources for the construction of meaning, and the design of the teaching environment. The learning-based instructional design theory is developed in response to the above requirements of constructivist learning. It provides new ideas and new ways of designing and transmitting teaching and learning to overcome the one-way transmission, passivity, and closure in traditional instructional design. This instructional design theory is particularly clear in the diagrammatic representation of the constructivist learning environment model proposed by David Jonathan, a contemporary advocate, and promoter of constructivist instructional design, as shown in Figure 3.

Blended learning has teacher-led student-led as its core idea, and both teacher teaching and student-led learning are extremely important. Constructivist learning theory also emphasizes the dual role of teaching and learning, which has a guiding role in the design of blended learning models. At the same time, constructivism attaches importance to the design of the teaching environment, and blended learning has rich multimedia and network technologies that can provide material support for the learning environment required by constructivism. Through the guidance of constructivist theory, the teaching objectives of blended learning can be effectively achieved and students' abilities in all aspects can be developed [19]. Once the particles of the classic PSO fall into the deep local optimum, it is difficult to escape. It has better ability to resist local optimal traps, so it has achieved a high precision of 3.82E-04. The researcher believes that with the development of mobile Internet technology and the improvement of information delivery tools, constructivist learning theory will continue to be further developed as the concept of blended learning is proposed and practiced and provides a broader space for putting it into practical application.

When teaching English as a foreign language in a blended learning environment, the first step for teachers is to present speaking tasks. Driven by the tasks, students can use their minds to use English actively by completing specific tasks and actively participate in various tasks to truly “learn by doing” and gain and accumulate corresponding learning experiences and enjoy the fun of learning. In the practical application of oral English teaching, task-based teaching makes students immerse themselves in the situation, truly gain emotional experience and adjust learning strategies, form a positive learning attitude, and promote the improvement of their practical language skills. The flexible use of cooperative learning in blended English speaking teaching activities can create a classroom atmosphere, stimulate students' interest in learning English, overcome classroom anxiety, and motivate students to actively exert their subjective initiative or maintain a high level of participation in online learning, increase the opportunities for students to use English to communicate and complete speaking learning tasks together, and improve students' speaking learning performance and speaking communicative competence.

**Figure 4:**
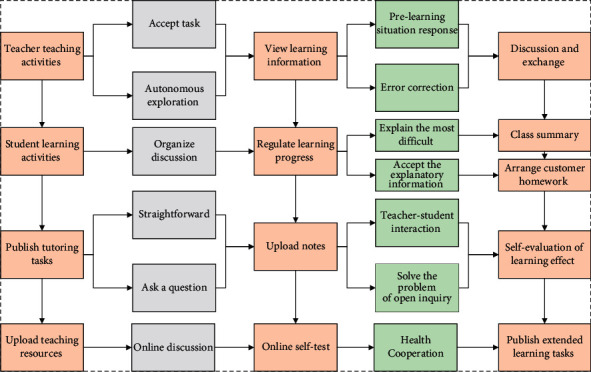
Model application design model.

Multiobjective optimization problems exist with multiple optimization objectives, and these subobjectives are often independent and conflicting with each other and cannot reach the optimal solution simultaneously. Theoretically, most optimal grouping processes are multiobjective problems, and solutions should be designed in a multiobjective framework. Based on the educational theory of collaborative learning, the basic principle of learning group formation is “intergroup homogeneity and intragroup heterogeneity,” where intergroup homogeneity ensures the success of all learning groups, while intragroup heterogeneity depends on different research topics and teachers' teaching needs. According to the specific requirements and principles in the proposed model of learning group formation, the multidimensional objective functions are set correspondingly. Due to the characteristics of the problem with many constraints, adding up the constraint violation degrees of all constraints may make the feasible domain of the algorithm smaller, which directly leads to few feasible solutions found and low reliability of the algorithm. Therefore, this study adopts the scheme of treating the constraint violation degree of each constraint as an objective separately, as shown in Figure 4.

In the real world, there do not exist any single-objective problems; in other words, single-objective problems are defined mainly for simplicity. This means that most of the time a person just chooses to consider the most important objective problem and ignores the others, thus converting a multiobjective problem into a single-objective problem. In addition, sometimes just one objective is chosen, and one or more other objectives are considered as constraints [20]. In both cases, the optimization process is simplified and redefined as single-objective optimization. Theoretically, most optimal grouping processes are multiobjective problems, and solutions should be designed in a multiobjective framework. Learning group formation as a multiobjective optimization problem is an essential and complex step in effective collaborative learning, and the purpose of this study is to propose a method based on a heuristic search strategy to enhance the intergroup homogeneity and intragroup heterogeneity of learning groups in collaborative learning environments, capable of grouping any number of prequalified learners with multiple characteristics into an arbitrary number of optimal inter- and intrahomogeneous groups [21].

Therefore, learning groups with smaller total mean deviation percentages are more favourable solutions. Through the information processing theory of absorption and addition, Whitlock proposes the information processing flow of the generative learning model: attention, motivation, prior knowledge and experience, and generation. The previous statement clearly shows that heterogeneity between groups and within groups is mainly reflected by values that reflect the average ability of all groups in a solution. Minimizing the overall error value of the learning group formula does not guarantee that the error values of all groups that make up the learners are minimized simultaneously. In other words, when the optimization algorithm is arranged to evaluate each learning group by its average fitness value, it is set to be insensitive to the fitness value of the constructing learners.

## 3. Results and Analysis

### 3.1. Performance Results of Improved Multiorganizational Particle Population Optimization Algorithm

**Figure 5:**
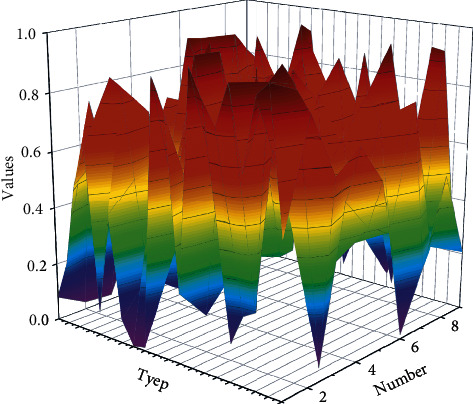
Comparison of the results of the tested algorithms on the benchmark function.

To compare the convergence speed, reliability, and successful performance of the algorithms, Figure 5 gives some key metrics, including the average number of objective function evaluations (FEs), success rate (SR), and success performance (SP) of each algorithm when it successfully achieves acceptable optimization accuracy. FEs and SRs are used to quantify the convergence speed and reliability of the algorithm. From Figure 5, TSLPSO converges fastest on 7 benchmark functions, followed by DLPSO (fastest convergence on 4 functions, i.e., functions F2, F6, F7, and F14), GL-PSO (fastest convergence on 3 functions, i.e., functions F1, F3, and F10), and L-SHADE (fastest convergence on 2 functions, i.e., functions F4 and F5). From the results of SR, TSLPSO is the most reliable with an average success rate of 100%, followed by DLPSO with a success rate of 98.62%.

From the convergence graph of single-peaked functions, the algorithm TSLPSO shows high convergence accuracy and fast convergence speed on most of the single-peaked functions except for the function F5. The convergence graphs of the multipeaked functions show that the algorithm TSLPSO not only converges with high accuracy but also converges faster than most of the algorithms. Moreover, DLPSO with only a dimensional learning strategy shows very competitive performance on these multipeaked functions. Thus, TSLPSO is generally effective for both single-peaked and multipeaked functions in terms of accuracy and convergence speed.

Most existing PSO algorithms use a linear weighting of individual optimal positions, random combination by dimension, or orthogonal combination of individual optimal positions and group optimal positions to construct learning examples, which guide the particle search instead of individual optimal solutions and group optimal solutions in the classical PSO velocity update formulation. This single-guidance learning mechanism can effectively avoid the oscillation phenomenon that is easily generated by the traditional PSO double-guidance action.

**Figure 6:**
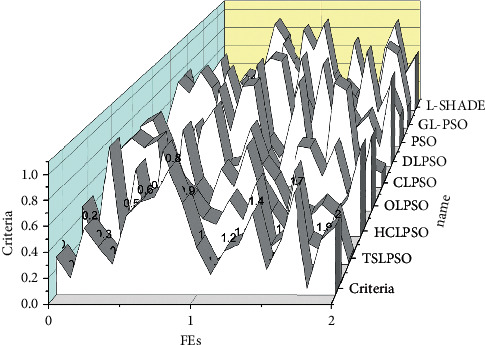
Graph of problem convergence curve.

However, the learning paradigms constructed by the above methods are highly stochastic and cannot ensure that the learning paradigm will not degenerate even when the individual optimal solution and the population optimal solution evolve every generation. Particles learning from degenerate paradigms is not conducive to maintaining the efficiency of the algorithm. The dimensional learning strategy proposed in this paper allows each dimension of the individual optimal position of the particle to learn from the corresponding dimension of the population optimal position respectively when constructing the paradigm, and the dimensional learning is formally incorporated into the learning paradigm only if it can improve the fitness of the paradigm, so that the dimensional learning strategy can avoid the phenomenon of degenerating the learning paradigm and the phenomenon of “two steps forward, one step back” phenomenon, as shown in Figure 6.

Although the learning paradigm constructed by dimensional learning can guide particles to search towards better regions, the fact that most of the particles are close to the population optimal position may cause premature convergence problems. To address this problem, we introduce an integrated learning strategy to enhance population diversity and help particles escape local extremes. TSLPSO alleviates the pressure of DLPSO adaptation evaluation by introducing dimensional learning subpopulations. Learners exhibit differences in learning behavior characteristics across resource presentations when learning online. In exploring document-based learning resources, this subsection focuses on three dimensions of learners' learning characteristics, namely, different learning knowledge types, course resource hotness, and course resource learning attainment, and the data visualization results are shown in Figure 6. During the study, the courseware resources that do not meet the procedural and descriptive knowledge characteristics are manually screened out, and only the courseware resources of these two major knowledge types are retained as the study objects.

### 3.2. Results of Applying the English Online Teaching Model

Adaptive follow-up assignments are personalized assignments on the Newton platform that take advantage of the instructional moment after proficiency learning to correct students' errors before they develop a basic understanding and before they move on to the next topic of study. Student work is completed to determine what students have mastered and understood and what they have not, and questions are selected from the Mastering Item Library to close individual gaps in understanding. Adaptive follow-up assignments also analyse student performance on proficiency learning tasks to select the types of assignments that are most helpful to students. Adaptive follow-up assignments are staged, with each set of questions based on the results of the previous set of questions. Promote the improvement of their language practical ability, and the flexible use of cooperative learning in mixed oral English teaching activities can create a classroom teaching atmosphere, stimulate students' interest in learning English, and overcome classroom anxiety. Students perform adaptive follow-up assignments until they complete the assigned problem set or master all concepts and material. In this way, students who lack practice and are late learners are required to complete as many as eight or more problem sets, while more advanced learners may be able to complete adaptive follow-up after one problem set. After the student completes the adaptive follow-up assignment, the student can see which questions he or she answered and how well he or she answered them. Adaptive follow-up assignments can be added to all proficiency learning tasks.

**Figure 7:**
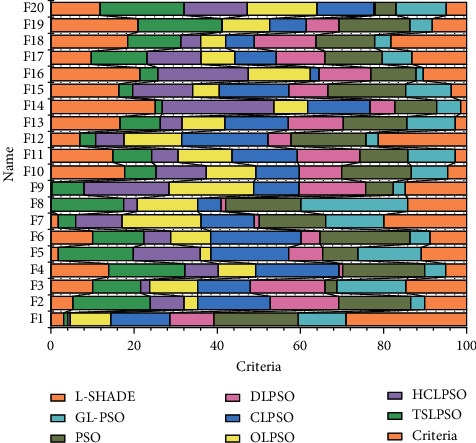
Analysis of video length differences.

The teacher first sets the number of problem sets the student receives, each of which takes about 15 minutes, but the specifics will vary from person to person; after this, a total point value is assigned to the assignment, and finally, a deadline is set and whether the student who performs well is expected to automatically receive full credit without taking the adaptive follow-up assignment. Adaptive follow-up assignments provide students with a personalized learning experience that helps each student continue to make progress in the course at the most appropriate time and in the best way possible. Overall, Newton's assignment resources can be categorized as fully adaptive assignments and partially adaptive assignments, as shown in Figure 7.

**Figure 8:**
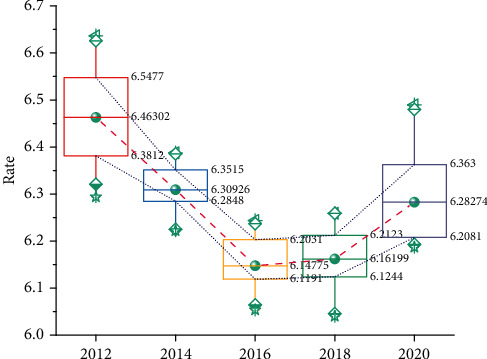
Pre- and postexperimental class results.

From Figure 7, it can be seen that in the resource hotness dimension, the mean of video resources above 10 min is higher than the mean of video resources between 5 and 10 min, and the mean of video resources between 5 and 10 min is higher than the mean of video resources between 0 and 5 min. Video resources above 10 min have a higher hotness H but a lower mean of resource learning attainment G, indicating that learners did not watch the whole video. The video resources of 0–5 min have a higher mean value than the mean value of course resource learning attainment G, although the hotness is lower, indicating that learners repeatedly watch the video resources. The resource designer can adjust the length of the video according to the purpose; for example, if the purpose is only to give learners a general understanding of the knowledge, then a video length of more than 10 min or 5–10 min can be used. If the purpose is to give learners knowledge, then a video length of 0–5 min can be used. Depending on the content of the video with or without the teacher and with or without subtitles, the learning characteristics of learners were explored in three dimensions: video resource presentation, course resource hotness, and course resource learning attainment, as shown in Figure 8.

The resources used in this round of action research were mainly topic-related lesson materials as well as microlearning resources. Before face-to-face teaching, teachers posted the resources in a WeChat class group for prelesson viewing. By posting interesting and targeted resources, students' interest in oral learning can be stimulated, and students can focus on the language points before class, so that they can be fully engaged in classroom activities with “excitement” and questions, thus improving students' communicative competence more effectively. Through WeChat's video forwarding function, video resources related to the background of the topic were released for students to watch before class to understand the linguistic situation of the language, which enriched students' language materials and made them understand and think about the topic before class, greatly improving the effect of face-to-face oral teaching. After class, dialogue practice assigned through the wing class network and dialogue drills are continued through the WeChat group cooperation group to achieve language review, consolidation, extension, and expansion and to cultivate students' good speaking habits of speaking and using English after class.

## 4. Conclusion

In the three rounds of action research, the researcher designed the content and teaching process of oral language teaching for the model and continuously reflected and optimized the implementation strategy and improved the teaching process in the teaching practice. The researcher conducted several questionnaires on the practical application effects of the model in speaking teaching, and after the longitudinal and cross-sectional pre- and posttest data comparison and analysis, it was found that after the experiment, the students in the experimental class had a greater improvement in their interest and attitude in English learning, cooperative communication ability, independent learning ability, evaluation awareness, and ability, problem-solving effect, and resource application effect in teaching compared to the students in the control class. This leads to the conclusion that using a blended learning-based speaking teaching model can effectively improve students' interest and attitude in English learning, can improve students' group cooperative communication and independent learning ability, can improve students' awareness and ability of evaluation, and can improve the effectiveness of problem-solving and the application of resources in teaching. Combining the DCOPSO algorithm with SVM, the traditional SVM hyperparameter search has the characteristics of poor globalization and low accuracy, while using the DCOPSO algorithm for search can solve these problems. The strategy enhances the diversity of the population and helps the population to jump out of local extremes. Finally, a two-population learning particle swarm optimization algorithm with heterogeneous learning strategies is proposed based on the dimensional learning strategy and the integrated learning strategy. One subpopulation uses a learning paradigm constructed by the dimensional learning strategy to guide the local search of particles, and the other subpopulation uses a learning paradigm constructed by the integrated learning strategy to guide the global search of particles. The two subpopulations achieve mutual collaboration in the search process through different information interaction mechanisms, which effectively improves the algorithm performance.

## Data Availability

The data used to support the findings of this study are available from the corresponding author upon request.

## References

[1] Sun Z., Anbarasan M., Praveen Kumar D. (2021). Design of online intelligent English teaching platform based on artificial intelligence techniques. *Computational Intelligence*.

[2] Yadav A., Vishwakarma D. K. (2020). A comparative study on bio-inspired algorithms for sentiment analysis. *Cluster Computing*.

[3] AbdulRahman S., Tout H., Ould-Slimane H., Mourad A., Talhi C., Guizani M. (2021). A survey on federated learning: the journey from centralized to distributed on-site learning and beyond. *IEEE Internet of Things Journal*.

[4] Jin S., Kim S., Kim H., Hong S. (2020). Recent advances in deep learning-based side-channel analysis. *ETRI Journal*.

[5] Kong X., Tong S., Gao H. (2020). Mobile edge cooperation optimization for wearable internet of things: a network representation-based framework. *IEEE Transactions on Industrial Informatics*.

[6] Hou R., Kong Y., Cai B., Liu H. (2020). Unstructured big data analysis algorithm and simulation of Internet of Things based on machine learning. *Neural Computing & Applications*.

[7] Ahmed Q., Raza S. A., Al‐Anazi D. M. (2021). Reliability-based fault analysis models with industrial applications: a systematic literature review. *Quality and Reliability Engineering International*.

[8] Song H., xiu-ying Han C. E., Montenegro-Marin C. E., krishnamoorthy S. (2021). Secure prediction and assessment of sports injuries using deep learning based convolutional neural network. *Journal of Ambient Intelligence and Humanized Computing*.

[9] Charbuty B., Abdulazeez A. (2021). Classification based on decision tree algorithm for machine learning. *Journal of Applied Science and Technology Trends*.

[10] Adi E., Anwar A., Baig Z., Zeadally S. (2020). Machine learning and data analytics for the IoT. *Neural Computing & Applications*.

[11] Marouf A. A., Hasan M. K., Mahmud H. (2020). Comparative analysis of feature selection algorithms for computational personality prediction from social media. *IEEE Transactions on Computational Social Systems*.

[12] Sun J., Tárnok A., Su X. (2020). Deep learning-based single-cell optical image studies. *Cytometry, Part A*.

[13] Liu C., Feng Y., Lin D., Wu L., Guo M. (2020). Iot based laundry services: an application of big data analytics, intelligent logistics management, and machine learning techniques. *International Journal of Production Research*.

[14] Pradhan K., Chawla P. (2020). Medical Internet of things using machine learning algorithms for lung cancer detection. *Journal of Management Analytics*.

[15] Onan A. (2021). Sentiment analysis on massive open online course evaluations: a text mining and deep learning approach. *Computer Applications in Engineering Education*.

[16] Ma L., Sun B. (2020). Machine learning and AI in marketing-connecting computing power to human insights. *International Journal of Research in Marketing*.

[17] Lee I., Shin Y. J. (2020). Machine learning for enterprises: applications, algorithm selection, and challenges. *Business Horizons*.

[18] Bui X. N., Nguyen H., Choi Y. (2020). Prediction of slope failure in open-pit mines using a novel hybrid artificial intelligence model based on decision tree and evolution algorithm. *Scientific Reports*.

[19] Sharafati A., Haji Seyed Asadollah S. B., Motta D., Yaseen Z. M. (2020). Application of newly developed ensemble machine learning models for daily suspended sediment load prediction and related uncertainty analysis. *Hydrological Sciences Journal*.

[20] Budhi G. S., Chiong R., Pranata I., Hu Z. (2021). Using machine learning to predict the sentiment of online reviews: a new framework for comparative analysis. *Archives of Computational Methods in Engineering*.

[21] Aleesa A. M., Zaidan B. B., Zaidan A. A., Sahar N. M. (2020). Review of intrusion detection systems based on deep learning techniques: coherent taxonomy, challenges, motivations, recommendations, substantial analysis and future directions. *Neural Computing & Applications*.

